# Knowledge, attitudes and practices of animal farm owners/workers on antibiotic use and resistance in Amhara region, north western Ethiopia

**DOI:** 10.1038/s41598-021-00617-8

**Published:** 2021-10-27

**Authors:** Kindu Geta, Mulugeta Kibret

**Affiliations:** 1 Department of Biology, Faculty of Natural and Computational Sciences, Debre Tabor University, Debre Tabor, Ethiopia P.O.Box 272,; 2 Department of Biology, Science College, Bahir Dar University, Bahir Dar, Ethiopia

**Keywords:** Microbiology, Diseases, Medical research, Risk factors

## Abstract

Inappropriate use of antibiotics in animal and human plays a role in the emergence and spread of bacteria resistant to antibiotics which threatens human health significantly. Although extensive use of these antibiotics could contribute to the development of drug resistance, information on the knowledge, attitude and practice of antimicrobial resistance and use among animal farm owners/workers in north western Ethiopia is rare. The objective of the present study was to assess knowledge, attitude and practice of animal farm owner/workers towards antibiotic resistance and use in Amhara regional state north western Ethiopia. A cross sectional study was conducted in selected cities of Amhara regional state from January to February, 2020. Data was collected from 91 participants using structured questionnaire and analyzed using SPSSS version 23. The results showed that 96.7% of respondents gave antibiotics to treat their livestock from different sources. Most of the respondents bought their antibiotics from private pharmacies without prescription and the most frequently mentioned antibiotics used to treat animal diseases was tetracycline (76.9%), followed by ampicillin (72.5%). Although, 90.1% of the animal farm owners heard about antibiotics and antibiotic resistance from different sources, they did not know the factors contributing to the transmission of resistant bacteria to humans and the impact of antibiotic resistance on human and animals’ health. Using the mean score 4.44 ± 0.15 as the cut-off, half of the animal farm owners/workers had good knowledge about antimicrobial resistance and use. 52.5% of animal farm owners/workers had positive attitudes towards wise antibiotic use and resistance with a mean score of 28.4 ± 0.5. However, 52.75% participants had poor practice with the mean score of practice 4.95 ± 0.17. Better knowledge, positive attitudes and better practices on antibiotic use and resistance were associated with farm owners/workers who engaged in higher education. Although poor awareness on antimicrobial resistance was perceived by 76.9% of respondents as very important factors that contribute to increasing antibiotic resistance, increasing the use of complementary treatments was perceived by the majority of respondents as very important strategies that contribute to reduce antibiotic use and resistance. The current study disclosed that there is low level of awareness among animal farm owners about the correct use of antibiotics and resistance. It is necessary to raise awareness, develop and implement interventions to reduce antimicrobial use and antibiotic resistance in the study area.

## Introduction

Antimicrobials are used in animal farming for a variety of reasons, including therapeutic, met phylaxis, prophylaxis, and growth promotion^1^. The indiscriminate use of antibiotics in food animal production has the potential to result in the development of antimicrobial-resistant bacteria strains by increasing selection pressure on bacteria to become resistant, as well as the presence of undesirable levels of drug residues in animal-derived food^2,3^. This will result in the accumulation of toxic and harmful residues in animal products, which will have a greater impact on consumer health^4,5^. Antibiotic use in food animals remains unregulated in many low-income countries, resulting in drug misuse and an increase in antibiotic resistance^6^. It is estimated that 25 million pounds of antimicrobials are used for non-therapeutic purposes in chickens, pigs, and cows, while only 3 million pounds are used for human medicine^7^. There is also evidence that a lack of antibiotic use and resistance education for farmers, limited awareness programs, and widespread antibiotic reliance are contributing factors to the problem's expansion^8^. The growing concern about the emergence of resistant bacteria to antimicrobials and their potential for transmission to humans through animal production has prompted various authorities around the world to implement measures to reduce antimicrobial use in livestock production^9–12^. Though some studies show the presence of naturally resistant bacteria, the widespread use of antimicrobial agents in animal production is suspected to be one of the major factors causing the emergence of antimicrobial resistance in bacterial strains^13–15^. There is ongoing pressure to limit the use of antimicrobials in animals in order to reduce the number of human infections caused by resistant bacteria transferred from animals^10,12^. Aside from the general public health implications, an increase in antimicrobial resistance, particularly to commonly used antimicrobials in livestock, may result in fewer treatment options as well as increased disease and production losses^15^.

Studies conducted in North and Northwest Ethiopia indicated that, the overall multi-drug resistance was 79.1% and 81.5% respectively^16,17^. Abebe et al.^18^ also conduct a study to isolate bacteria from poultry wastes and test their antimicrobial susceptibility patterns. The authors indicated that all bacterial isolates demonstrate multi-drug resistant for tested antimicrobials. A study conducted by Leopold et al.^19^ also found a high level of resistance to the commonly used antibiotics in the sub Saharan African region.

Several high-income countries are now keeping an eye on trends in antimicrobial use (AMU) and antimicrobial resistance (AMR) in livestock^20^. However, these trends are generally scarce, particularly in low and middle-income countries^2^. Ethiopia has a large livestock population, with 60.4 million cattle, 31.3 million sheep, 32.7 million goats, and 1.4 million camels^21^. Regulations on AMU in livestock in Ethiopia, like in many other developing countries, are poorly enforced, and farmers have easy access to veterinary drugs. In Ethiopia, information on AMU and resistance in animals is currently scarce. Knowledge, attitudes, and practices, in particular, are factors that contribute to the use of antimicrobials and resistance, and intervention to reduce antibiotic resistance and use in animals on farms, as well as the impact of antimicrobial resistance, are poorly understood. Information on farm owners' and workers' knowledge, attitudes, and practices (KAP) regarding antimicrobial resistance and use will aid in the development of strategies to maximize and preserve the benefits of AMU in animal production while posing the least risk to public health. As a result, a study was conducted in selected cities of Amhara regional state, North western Ethiopia, on the knowledge, attitude, and practice of animal farm owners/workers regarding antimicrobial use and resistance.

## Materials and methods

### Study design, period and setting

**Figure 1:**
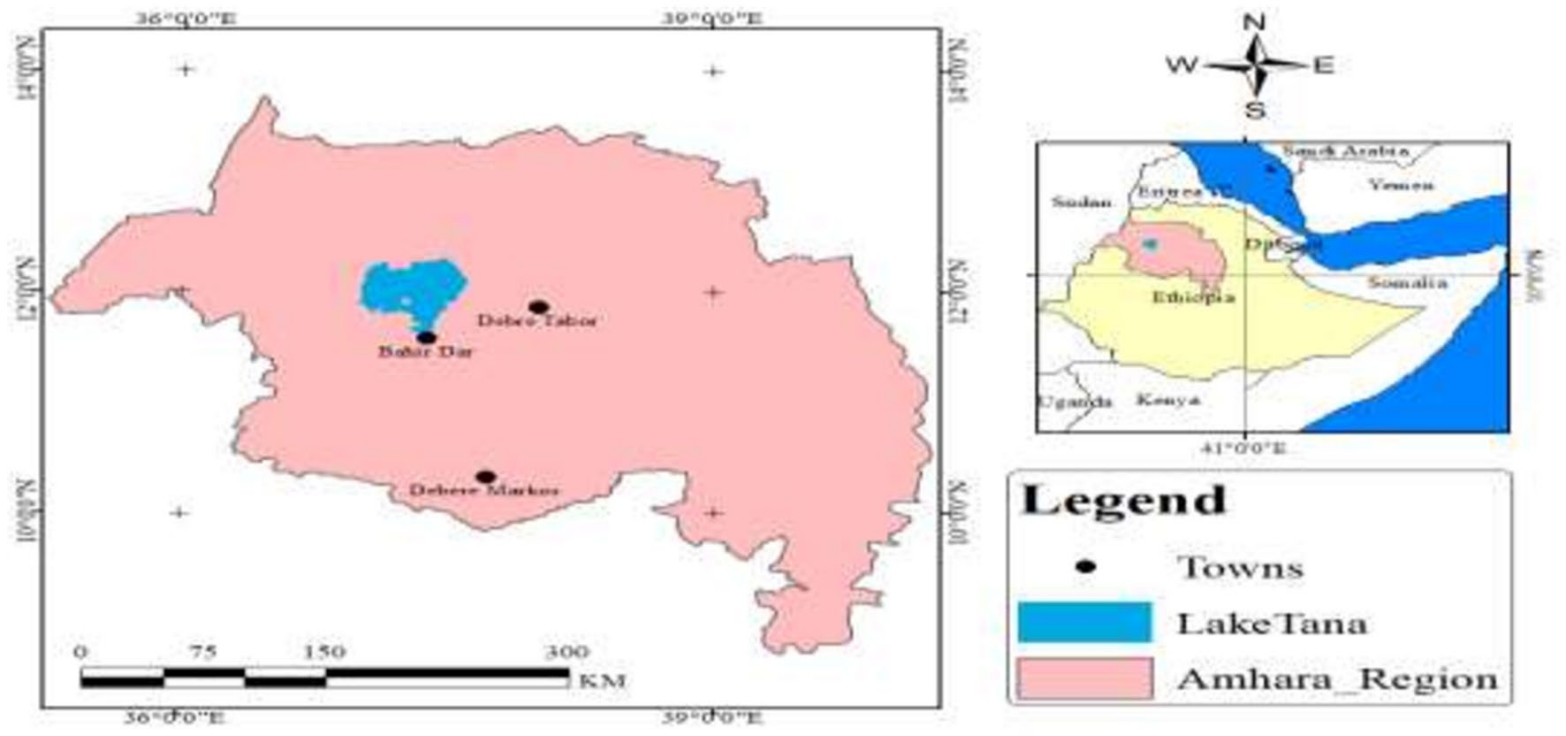
Map of the study area. ArcGIS software version 10.5, https://njuclrd.weebly.com/arcgis-105-crack-free-download.html.

A cross-sectional survey study was conducted among eligible animal farm owners/workers working in animal farms from November 2019 to February 2020. Debre Markos, Debre Tabor and Bahir Dar cities were selected using systematic random selection method (Fig. 1). At the time of the survey, 805 animal farm owners and workers were working in a total of 288 animal farms^22^.

### Sample size and sampling technique

A multi stage sampling procedure was applied to select respondents in the region. First three cities were randomly selected from Amhara region. From these cities, 30 animal farms were systematically selected from the study areas. Secondly, animal farms were stratified in to poultry, beef cattle and dairy farms. A total of 91 participants were allotted for animal farm owners/workers. Then the participants were proportionally allocated to each farm (poultry = 26, beef cattle = 11 and dairy farms = 54) based on the population they have and finally study subjects were selected by simple random sampling technique.

Animal farm owners/workers who worked for less than 6 months in selected livestock farms during the study period and give service during the study period but unwilling to participate in the study were excluded.

### Data collection tools and procedure

Structured questionnaires were developed by the researchers after performing a thorough literature review of comparable studies^23,24^ to collect the data. The questionnaire was prepared in English and translated into Amharic. The questionnaire was then reviewed and assessed by subject experts for its content, design, relevance and understanding. After assessment of its content relevancy and appropriateness, it was confirmed by five clinical pharmacy lecturers, three medical microbiology professors and two veterinary microbiologists at Bahir Dar and Debre Markos universities and minor modifications were made based on their comments. In addition, quantitative content validity of the instrument was determined based on Lawshe’s content-validity ratio (CVR)^25^. CVR was calculated for every item, and all items had a CVR scores of a minimum of 0.71. Items with CVR ≥ 0.7 are acceptable, and if an item doesn't reach this threshold, it's ordinarily deleted from the ultimate instrument^26^. Then, a content-validity index (CVI) was obtained by calculating the mean of the CVR values for all items meeting the CVR threshold of 0.7 and reserved for the ultimate instrument. the ultimate reported content validity–index value for the instrument was 0.73, indicating acceptable content validity^27^.

Moreover, for reliability a pilot study was done on 10% of the study population who were excluded from the final analysis and necessary changes made accordingly. Data collectors were trained and monitored on a regular basis by the investigators. Reliability analysis of the instrument was executed by calculating Cronbach’s α. The α-value of the questionnaire was 0.78, indicating acceptable internal consistency.

The questionnaires contain seven parts. In the first part, the demographic data such as age, sex, religion, marital status, type of farm and level of education were reported. In the second part, each individual was asked to answer a number of questions on antibiotic uses for their animals. The third and fourth parts of the questions are concerned on the knowledge and attitudes of participants on antibiotics use and resistance. The fifth part of questions concerned on participants practices on antibiotic uses. In the sixth parts of the questionnaire, participants were asked about factors contributing to increasing antibiotic resistance. In the last parts of the questionnaire, participants were asked about the possible measures that can be applied to decrease the risk of antibiotic resistance.

Three trained data collectors (animal health professionals) were assigned for the data collection process. For the study participants, hard copies of written informed consent containing questionnaires were distributed. Then, data collectors collected completed questionnaires from study participants, and the questionnaires were checked for accuracy. Incomplete questionnaires were returned to participants in the study for completion. After completion, the questionnaires were labeled and coded using questionnaire number.

### Methods of measurement (scoring)

For knowledge and practice assessment, each correct response was given a score of 1 while a wrong or doubtful response was scored as 0. Responses to attitude-related questions were graded on a 3-point Likert scale, with a ‘1’ for disagree and a ‘3’ for agree. We used the mean as a cut-off point because there was no cut-off point to assess poor and better knowledge. Scores above and equal to the mean would indicate better knowledge, practice, and a positive attitude, whereas scores below the mean would indicate a lack of knowledge, practice, and a positive attitude.

### Data quality control and validation

Data collection tools were pre tested on 10% of the study population who were not included in the final study to for validation of the data collection instrument. The contents of the data collection tools were slightly modified based on the pilot survey, and suggestions from various people were included. Data collectors were trained and monitored on a regular basis by the investigators.

### Statistical data analysis

The response alternatives for knowledge and practice items were dichotomous. The questions on attitude used Likert-style responses. Data was entered and analyzed using the Statistical Package for Social Sciences (SPSS 23.0, USA). Normality of data was tested using Kolmogorov–Smirnov test. One Way ANOVAs was used to compare the mean scores of knowledges, attitude and practice of respondents. The Chi-square test was used to assess the relationship between AMR knowledge, attitude, and practice and independent variables. A multivariate linear regression model was used to identify factors associated with good antibiotic resistance and use knowledge, practice, and attitudes. A P value of 0.05 (two sided) was used to determine statistical significance. Finally, the analyzed data were organized and presented in the appropriate tabular, graphical, and narrative formats.

### Ethical consideration

This study was approved by the Research and Ethical Review Board of Bahir Dar University College of Science with reference number PGRCSVD/17/2019. Moreover, written informed consent was obtained from all farm owners/workers and all research was performed in accordance with relevant guidelines/regulations.

## Results

### Soci-demographic data

Investigation of demographic parameters showed that majority of the participants were male (86.8%), married (61.5%) and at the age of 31–45 years (41.8%). Among the 91 respondents who determined their level of education, most (30.8%) participants were completed secondary school. Most participants were orthodox (94.5%) and animal farm owners (81.3%). Most of the respondents (86.8%) had 1–100 animals and 54 (59.3%) of the them had dairy farms (Table 1).

**Table 1:** Demographic characteristics of animal farm owners/workers (N = 91).

Variables	Categories	N	%
Sex	Male	79	86.8
	Female	12	13.2
Age	18–30	24	26.4
	31–45	38	41.8
	46–55	18	19.8
	56–65	11	12.1
Marital status	Single	27	29.7
	Married	56	61.5
	Divorce	8	8.8
Level of education	Uneducated	6	6.6
	Primary	19	20.9
	Secondary	28	30.8
	Diploma	16	17.6
	Technique	1	1.1
	University	21	23.1
Religion	Orthodox	86	94.5
	Protestant	5	5.5
Type of farm	Poultry	26	28.6
	Dairy	54	59.3
	Beef	11	12.1
Stakeholder	Owner	74	81.3
	Son	2	2.2
	Worker	15	16.5
No of animals	1–100	79	86.8
	101–200	1	1.1
	201–300	3	3.3
	301–400	1	1.1
	500 +	7	7.7

### Antibiotic use

The majority of respondents (96.7%) gave antibiotics to treat their livestock from different sources. Most of them bought the antibiotics from private pharmacy without prescription, some of them provided from clinical veterinary services and others obtained the antibiotics from previously stored in their house (Table 2).

**Table 2:** Antibiotic use for animals in north western Ethiopia by animal farm owners/workers (N = 91).

Questions	Responses	N	%
Have you given antibiotics for your animals?	Yes	88	96.7
	No	3	3.3
Where did you obtain the antibiotics that you gave for your animals?	From veterinarian prescription	17	18.7
	Without prescription from private pharmacy	66	72.5
	Left over from a previous course	5	5.5
	None	3	3.3
Why you used antibiotics for your animal without prescription?	Minimize cost	28	30.8
	Previous experiences	31	34
	Quick relief	10	11
	Lack of time	22	24.2
How many times have you give antibiotics for your animals per a month?	Never	3	3.3
	Once	4	4.4
	2–5 times	66	72.5
	More than 5 times	18	19.8

When animal farm owners/workers in the study area were asked why they used antibiotics for their animal without prescription, most of the respondents (34.1%) said that they had previous experience to use the antibiotics. Regarding frequency of antibiotic use 72.5% of respondents gave antibiotic for their animals 2–5 times per month (Table 2).

**Figure 2:**
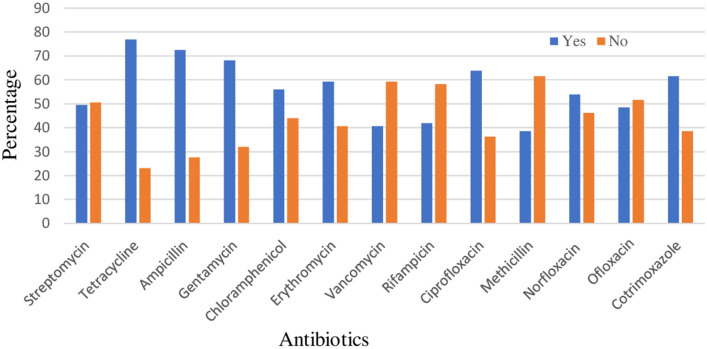
Common antibiotics used by animal farm owners/workers.

Most of the respondents were able to mention the brand name of at least one antibiotic that they had mostly administered to their animals. Tetracycline, ampicillin, gentamycin, ciprofloxacin and cotrimoxazole were the most frequently used groups of antibiotics respectively (Fig. 2).

### Antibiotic resistance

**Figure 3:**
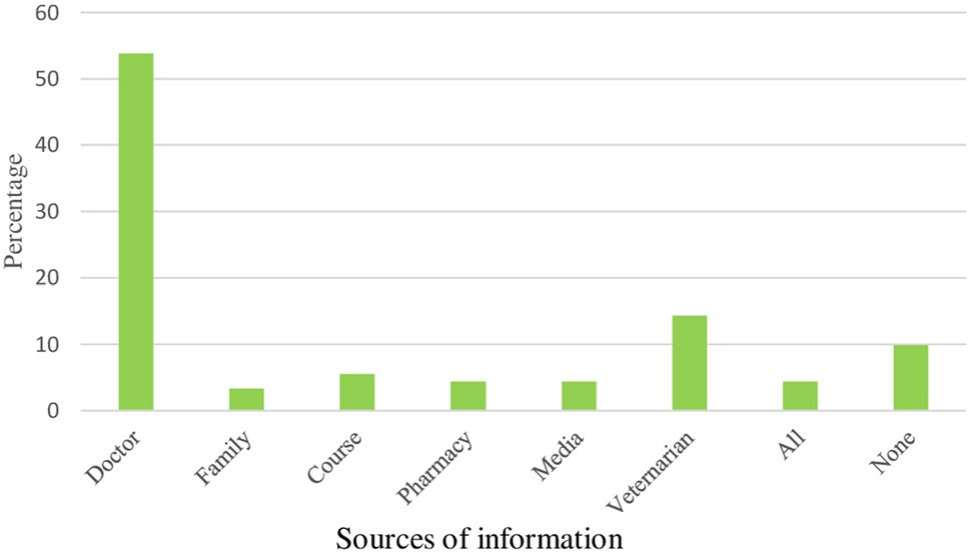
Sources of information on ABR and use.

It was found that a large proportion of the animal farm owners (90.1%) participating in the survey heard about antibiotics and antibiotic resistance from different sources. Most respondents heard this information from doctors/nurses (53.8%), whereas only 3.3% of respondents heard the information from families (Fig. 3).

**Figure 4:**
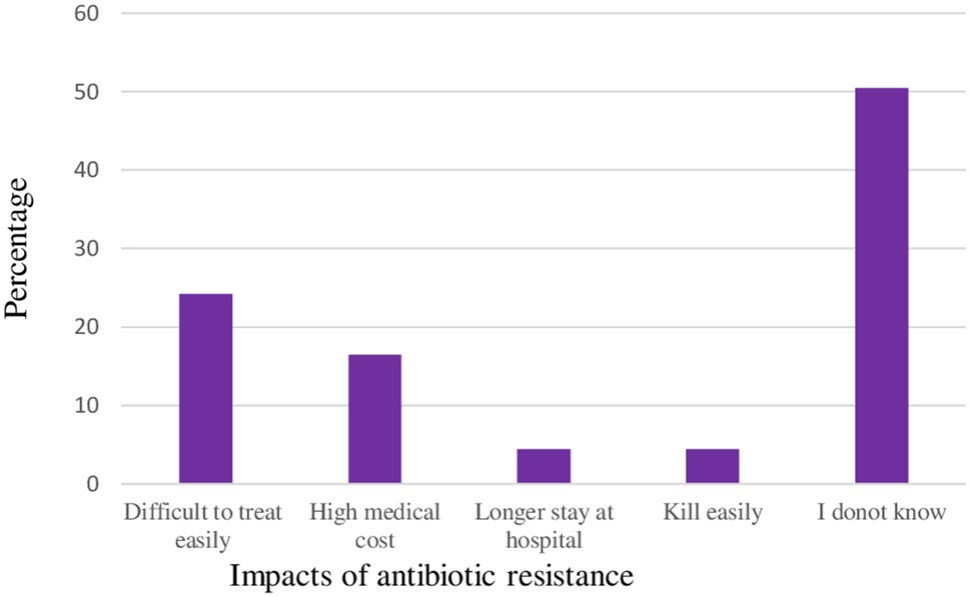
Impacts of antibiotic resistance.

When animal farm owners in the study area were asked if they knew any possible impacts of antibiotic resistance on human and animals’ health, most (24.2%) of respondents responded that difficult to treat disease caused by resistant microorganism easily was the possible impact of AMR. On the other hand, 50.5% of the respondents did not know the impact of antibiotic resistance on human and animals’ health (Fig. 4).

**Figure 5:**
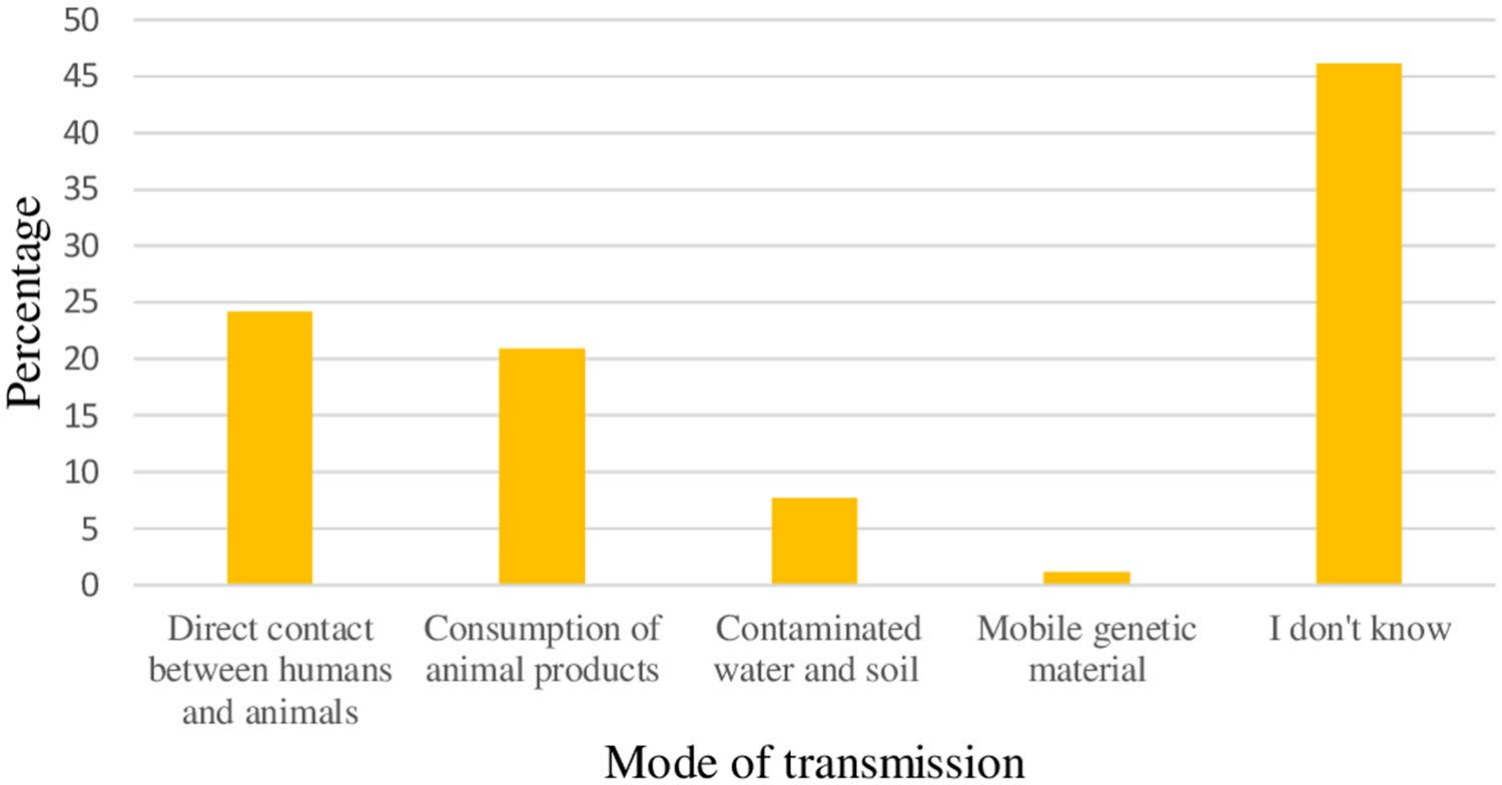
Mode of transmission of resistant bacteria to humans.

From the survey, it was found that 24.2% of the respondents responded that the main mode of transmission of resistant bacteria to the humans was through the direct contact between humans and animals. On the other hand, 46.2% of the respondents considered that they did not know factors that contributed to the transmission of resistant bacteria to humans (Fig. 5).

### Knowledge of animal farm owners/workers on AMR and use

As indicated in Table 3, the mean knowledge score was 4.44 ± 0.15. Using the mean score as the cut-off, half of the livestock farm owners/workers (50.5%) had good knowledge about ABR and use. Most farm owners/workers 59.3% responded correctly as antibiotics can be used for all types of diseases in animals and 58.2% of them knew that antibiotics are harmful for beneficial bacteria living in the body. Additionally, 57.1% of the farm owners/workers were aware that antibiotics have side effects. However, a relatively low proportion of the respondents (42.9%) were aware that improper use of antibiotics in animal farm can cause AMR (Table 3).

**Table 3:** Knowledge of animal farm owners/workers towards antimicrobial resistances and use in Amhara regional state, North Western Ethiopia (N = 91).

Knowledge related items	Correct N (%)	Incorrect N (%)
Antibiotics can be used in animals for weight gain	51 (56)	40 (44)
Antibiotics can be used for all types of diseases in animals	54 (59.3)	37 (40.7)
All commercial antibiotics show the same curative effect in animal diseases	50 (54.9)	41 (45.1)
Improper use of antibiotics in animal farm can cause AMR	39 (42.9)	52 (57.1)
Antibiotics are not harmful for beneficial bacteria living in the body	53 (58.2)	38 (41.8)
All antibiotics I used can cure animals	54 (59.3)	37 (40.7)
Antibiotics have side effects	52 (57.1)	39 (42.9)
Bacteria can become resistant against antibiotics	51 (56)	40 (44)

Overall level of knowledge	Frequency (%)	
Better	46 (50.5)	
Poor	45 (49.5)	

The mean knowledge scores of our study had a significant correlation with the level of education of respondents (Table 4). Based on multiple linear regression analysis, having lower level of education were significantly associated with lower level of knowledge (P < 0.05). Those study participants who had higher level of education (university) had 0.352 times better knowledge than uneducated participants (95% CI 0.18–0.53) (Table 5).

**Table 4:** Relation between participants’ characteristics and mean scores of knowledges, attitude and practices on antibiotic resistance and use.

Demographic variables	Categories	Knowledge score		Attitude score		Practice score	
		Mean ± SD	P-value	Mean ± SD	P-value	Mean ± SD	P-value
Sex	Male	4.5 ± 1.4	0.615	28.9 ± 4.6	0.02	5.4 ± 2.4	0.054
	Female	4.3 ± 1.4		25.4 ± 5.3		3.9 ± 2.4	
Age	15–30	4.1 ± 1.3	0.225	27.3 ± 5.1	0.029	4.4 ± 2.5	< 0.0001
	31–45	4.4 ± 1.5		27.5 ± 4.6		4.7 ± 2.3	
	46–55	4.4 ± 1.3		30.2 ± 4.5		5.7 ± 1.9	
	56–65	5.2 ± 1.2		31.2 ± 4.1		7.8 ± 1.5	
Marital status	Single	4.3 ± 1.4	0.328	27.8 ± 5.2	0.049	4.7 ± 2.5	0.024
	Married	4.4 ± 1.4		28.2 ± 4.6		5.1 ± 2.4	
	Divorce	5.1 ± 1.0		32.4 ± 4.8		7.4 ± 2.5	
Level of education	Uneducated	3.0 ± 0.6	0.001	22.8 ± 2.2	< 0.0001	4.5 ± 2.3	< 0.0001
	Primary	3.7 ± 1.5		27.2 ± 4.3		4.6 ± 2.3	
	Secondary	4.5 ± 1.0		26.5 ± 4.2		4.1 ± 2.0	
	Diploma	4.8 ± 1.6		30.0 ± 4.7		5.1 ± 2.8	
	Technique	6.0		35.0		9	
	University	5.1 ± 1.2		32.2 ± 3.4		7.3 ± 1.3	
Religion	Orthodox	4.5 ± 1.4	0.086	28.5 ± 4.8	0.337	5.1 ± 2.5	0.449
	Protestant	3.4 ± 1.1		26.4 ± 5.4		6 ± 1.9	
Type of farm	Poultry	4.85 ± 1.3	0.046	28.7 ± 4.6	0.254	5.3 ± 2.5	0.250
	Dairy	4.4 ± 1.5		28.7 ± 5.0		5.3 ± 2.3	
	Beef	3.6 ± 0.9		25.9 ± 4.2		3.9 ± 2.7	
Stakeholder	Owner	4.6 ± 1.4	0.220	28.7 ± 4.8	0.461	5.4 ± 2.4	0.156
	Son	4.5 ± 2.1		25.5 ± 2.1		3.0 ± 1.4	
	Worker	3.7 ± 1.2		27.5 ± 5.1		4.4 ± 2.6	
No of animals	1–100	4.6 ± 1.4	0.841	28.5 ± 4.9	0.678	5.2 ± 2.4	0.437
	101–200	5.0		35		7	
	201–300	5.0 ± 0.0		26.7 ± 1.5		2.7 ± 1.5	
	301–400	4.0		29		5	
	500 +	4.0 ± 1.2		27.9 ± 5.7		5.4 ± 3.2

**Table 5:** Multiple linear regression analyses for predicting score of knowledge, attitude and practice.

Variables	Factors	*B*	t	Sig.	95.0% Confidence Interval for *B*	
					Lower bound	Upper bound
Knowledge	Constant	3.725	8.349	< 0.0001	2.838	4.613
	Education	0.352	4.014	< 0.0001	0.178	0.527
Attitude	Constant	21.815	15.674	< 0.0001	19.046	24.583
	Age	1.887	2.735	0.008	0.515	3.260
	Education	1.615	5.897	< 0.0001	1.070	2.159
Practice	Constant	1.755	2.513	0.014	0.366	3.143
	Age	1.439	4.157	< 0.0001	0.750	2.127
	Education	0.655	4.771	< 0.0001	0.382	0.928

### Attitude of animal farm owners/workers

Most of the farm owners/workers (52.5%, *N* = 48) had positive attitudes on appropriate antibiotic use and resistance with a mean score of 28.4 ± 0.5. Using the mean score as the cut-off, majority (72.5%) of the respondents strongly agreed that, antimicrobial usage for protection against diseases on farms is the most important, purchasing of AMD from a drug company or cooperative with a legal permit is safe (70.3%), antibiotic resistance in animals is not important for public health (64.8%) and usage of the same AMD for long period of time can lead to AMR (61.5%). However, a relatively low proportion of the respondents (25.3%) were strongly agreed that the most important reason for choosing AMD on my farm is its effectiveness, there is relationship between antibiotic use in animals and development of resistance (36.3%), the use of antibiotics in animals causes the emergence of resistant bacteria which cause diseases in humans (38.4%) and restriction of antibiotic use in animals will lead more benefit than damage (40.7%) (Table 6).

**Table 6:** Attitude of animal farm owners/workers towards antimicrobial resistances and use in Amhara regional state, North Western Ethiopia (N = 91).

Attitude related items	Agree N (%)	Neutral N (%)	Disagree N (%)
Antibiotic resistance in animals is not important for public health	59 (64.8)	14 (15.4)	18 (19.8)
There is relationship between antibiotic use in animals and development of resistance	33 (36.3)	19 (20.8)	39 (42.9)
The use of antibiotics in livestock causes the emergence of resistant bacteria which cause diseases in humans	35 (38.4)	16 (17.6)	40 (44)
Restriction of antibiotic use in animals will lead more benefit than damage	37 (40.7)	9 (9.9)	45 (49.4)
Use of antibiotic in animals does affect myself or my family indirectly	47 (51.6)	10 (11)	34 (37.4)
AMD residues and drug resistance will occur when AM are not used prudently	47 (51.6)	16 (17.6)	28 (30.8)
Antimicrobial usage for protection against diseases on farms is the most important	66 (72.5)	8 (8.8)	17 (18.7)
Usage of the same AMD for long period of time can lead to AMR	56 (61.5)	9 (9.9)	26 (28.6)
Usage of AMD for non-therapeutic reasons lead to AMR	49 (53.8)	18 (19.8)	24 (26.4)
Purchasing of AMD from a drug company or cooperative with a legal permit is safe	64 (70.3)	9 (9.9)	18 (19.8)
The most important reason for choosing AMD on my farm is its effectiveness	23 (25.3)	9 (9.9)	59 (64.8)
Sale and distribution of AMD shall only be done by persons permitted to do so by law	49 (53.8)	20 (22)	22 (24.2)
Drug withdrawal periods should be adhered to as per the prescription to avoid drug residues in meat or animal products	51 (56)	15 (16.5)	25 (27.5)

Overall level of attitude	Frequency (%)		
Positive	48 (52.8)		
Negative	43 (47.2)		

Mean attitude of our study was significantly varied across sex, age groups and levels of education (Table 4). Multiple linear regression analysis showed that age and having lower level of education were significantly related with having negative attitude (P < 0.05) (Table 4). Results demonstrated that age and level of education were positively correlated with increased level of attitude on antibiotics use and resistance. Those study respondents who had higher level of education (tertiary) had 1.62 times positive attitude than uneducated participants (95% CI 1.07–2.16) and participants at the age group of 56–65 had 1.89 times positive attitude than the age group of 18–30 years (95% CI 0.52–3.26) (Table 5).

### Practice of animal farm owners/workers

The highest number of participants 55 (60.4%) were responded correctly the statement that when animals get sick, I use my antibiotics before consulting a veterinarian and even if I knew unconscious antibiotic use will be give any harm to public health, I would continue to use antibiotics in animals while the least number of participants 83 (35.8%) were respond correctly on the statement I am ready to go for laboratory test before choosing antimicrobial drugs for use of my animals (42.9%) (Table 7).

**Table 7:** Practice of animal farm owners/workers towards antimicrobial resistances and use in Amhara regional state, North Western Ethiopia (N = 91).

Practice related items	Correct N (%)	Incorrect N (%)
When animals get sick, I use my antibiotics before consulting a veterinarian	55 (60.4)	36 (39.6)
I do not consult a veterinarian to ask whether I need to use antibiotics or not	58 (52.7)	43 (47.3)
I do not read the prospectus before using antibiotics	43 (47.3)	48 (52.7)
I increase the dose of antibiotics and frequency of administration as long as animals do not show any signs of recovery	49 (53.8)	42 (46.2)
If animals feel better after the first day of treatment, I stop giving the antibiotics	45 (49.5)	46 (50.5)
I consider the recommendations of other farmers about antibiotic use	43 (47.3)	48 (52.7)
Even if I knew unconscious antibiotic use will be give any harm to public health, I would continue to use antibiotics in animals	55 (60.4)	36 (39.6)
I am ready to go for laboratory test before choosing antimicrobial drugs for use of my animals	39 (42.9)	52 (57.1)
Farmers adhere to specified drug withdrawal periods before sending animals to the slaughterhouse	44 (48.4)	47 (51.6)
Farmers don’t sell animal products which have been treated with antimicrobial drugs	51 (56)	40 (44)

Overall level of practice	Frequency (%)	
Better	43 (47.25)	
Poor	48 (52.75)	

The mean score of the participant’s practice was 4.95 ± 0.17. Participants who scored below the mean score were 48 (52.75%) which was considered poor practice) and above the mean score were 43 (47.25% which was considered good practice). Practice scores significantly varied across age groups, marital status, and levels of education (Table 4).

Results demonstrated that age and education levels were positively correlated with increased levels of practice on antibiotics use and resistance. Those study participants who had higher level of education (tertiary) had 0.66 times good practice than uneducated participants (95% CI 0.38–0.93) and participants at the age group of 56–65 had 1.44 times good practice than the age group of 18–30 years (95% CI 0.75–2.13) (Table 5).

### Associations between KAP

Pearson’s correlation was used to assess the bivariate relationship between KAP scores. KAP scales were all significantly (P < 0.05) and positively correlated with the strongest correlations between knowledge and attitudes (0.36) and knowledge and practices (0.31). Practice was also positively and significantly correlated with attitudes (0.46) (Table 8).

**Table 8:** Correlation between KAP.

Variables	Knowledge	Attitude	Practice	N
**Knowledge**				
Pearson correlation	1	0.365**	0.307**	91
Sig. (2-tailed)		< 0.0001	0.003	
**Attitude**				
Pearson correlation	0.365**	1	0.455**	91
Sig. (2-tailed)	< 0.0001		< 0.0001	
**Practice**				
Pearson correlation	0.307**	0.455**	1	91
Sig. (2-tailed)	0.003	< 0.0001		

**Correlation is significant at the 0.01 level (2-tailed).

### Factors that contribute to increase antibiotic resistance

**Figure 6:**
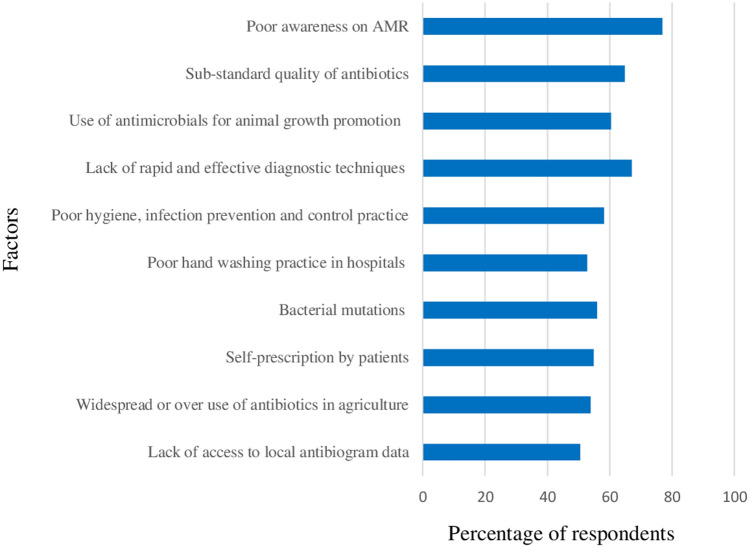
Factors that contribute to increase antibiotic resistance.

Although most factors were significantly perceived by the majority of respondents as very important factors that contribute to increasing of antibiotic resistance, poor awareness on AMR (76.9%), lack of rapid and effective diagnostic techniques (67%), sub-standard quality of antibiotics (64.8%) and use of antimicrobials for animal growth promotion (60.4%) were the most important factors (Fig. 6).

### Interventions that contribute to reduce antibiotic use and resistance

**Figure 7:**
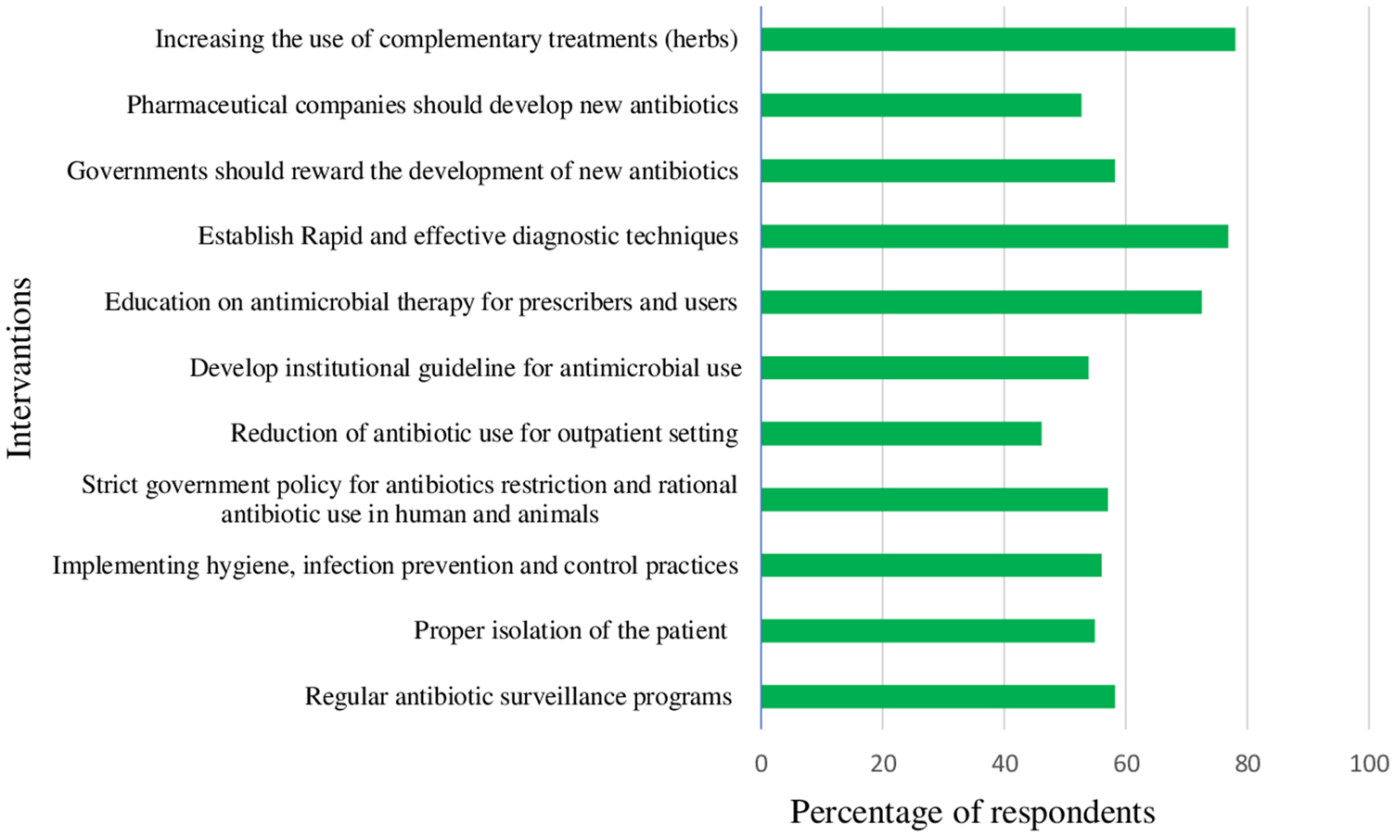
Interventions that contribute to reduce antibiotic use and resistance.

Although most strategies were perceived by the majority of respondents as very important strategies that contribute to reduce antibiotic use and resistance, increasing the use of complementary treatments (herbs) (78%), establish rapid and effective diagnostic techniques (76.9%) and education on antimicrobial therapy for prescribers and users (72.5%) were the most important strategies (Fig. 7).

## Discussion

Inappropriate antibiotic use in animal production has serious consequences for public health and the environment^28,29^. MDR organisms have been isolated from livestock, livestock environments, and employees, posing serious public health threats, particularly in low- and middle-income countries (LMICs) such as Ethiopia^30–33^. These MDR bacteria are frequently transmitted to humans via the food chain, the environment, bodies of water, and close contact with these animals^28,34,35^.

In the current study, the majority of animal farm owners/workers used antibiotics in their farms in the previous month. For financial reasons, animal farm owners/workers did not seek veterinarian advice before administering antibiotics to their animals, and the majority of them (72.5%) obtained antibiotics from a private pharmacy without a prescription. Farmers in many countries can easily obtain antibiotics without a prescription^36,37^ to reduce the cost of veterinary services and use their prior experience to use the antibiotics, which supports our findings. According to an Indian study, only one-third of farmers seek the assistance of a veterinarian to reduce veterinary costs^36^. Although a number of measures have recently been implemented to limit antibiotic use in human medicine in order to combat antibiotic resistance, their application to the field of animal health is moving slowly and insufficiently. As a result, animal farm owners can still easily obtain antibiotics from veterinary clinics without a prescription. Tetracycline, ampicillin, cotrimoxazole, gentamicin, and quinolone-based antibiotics were the most commonly used to treat animal diseases in the current study. This is similar to the findings of Adebowale, Adeyemo^38^ and Ogunleye, Oyekunle^39^ who found that gentamicin, tetracycline, quinolones, and sulfonamides were the most commonly used antibiotics in poultry in Ogun state. A number of other studies have estimated antibiotic use and resistance in livestock. Due to their low cost and ease of availability, gentamicin and tetracycline were the most commonly reported antibiotics by farmers^40^. Misuse and resistance to quinolones (ciprofloxacin) in animals is particularly concerning because ciprofloxacin is one of the essential medicines listed for humans^41^.

Despite the fact that the majority of animal farm owners/workers had heard about antibiotics and ABR from various sources, there were significant gaps in their understanding of antibiotic resistance and use. Some farm owners/workers believed antibiotics could be used to treat all types of diseases in animals, and only 42.9% understood that improper antibiotic use in animal farms can lead to AMR. This finding was lower than the finding reported by Ozturk and Celik^42^ who found that 72% of farmers believed that antibiotic misuse caused the spread of resistant bacteria. Nuangmek, Rojanasthien^43^ also reported that two thirds of respondents agreed with the right statements for inappropriate use of antimicrobial drugs can cause antimicrobial resistance. This is frequently demonstrated by the fact that the majority of farmers were aware that bacteria could become resistant to antibiotics but were unaware of the mode of transmission and its impact on animal and human health.

On the other hand, antibiotics were said to have no side effects by 42.9% of farm owners/workers This result was consistent with a previous report by Ozturk, Celik^42^ who found that nearly 38 percent of farmers believed antibiotics had no side effects. The knowledge levels of older farm owners/workers were significantly higher than those of younger respondents. This could be due to the years of hands-on experience. Animal farmers with tertiary education knew and used ABR more than those with primary education.

The majority (52.8%) of animal farm owners/workers had a positive attitude toward ABR and use, with a mean score of 28.4 ± 0.5. Most farm owners/workers agreed that antimicrobial use for disease prevention on farms is the most important, purchasing AMD from a drug company or cooperative with a legal permit is safe, and that ABR in animals is not important for public health. On the other hand, 64.8% disagreed that the most important reason for using AMD on their farm is its effectiveness rather than its economic cost. This finding was supported by the finding of Nuangmek, Rojanasthien^43^ who reported that 63.7% of the respondents incorrectly believed that the primary reason for using antimicrobials on the farm are the economic costs and benefits.

A study conducted in Thailand reported that nearly two-thirds of respondents agreed with the correct statements that long-term use of similar antimicrobial drugs can lead to antimicrobial resistance, and nearly 40% of all respondents believed that using antimicrobial drugs for non-therapeutic reasons (as a growth promoter) or as a prophylactic treatment cannot cause antimicrobial resistance which supports our finding^43^.

Moreover, only 36.3% of respondents correctly believed that there is a link between antibiotic use in animals and the development of resistance, while 38.4% believed that antibiotic use in livestock causes the emergence of resistant bacteria that cause diseases in humans. The attitudes of older farm owners/workers were significantly higher than those of younger participants. This could be due to the years of experience. Farm owners with a secondary education were more optimistic about antibiotic resistance than those with a tertiary education (Table 4).

With a mean score of 4.95 ± 0.17, 47.25% of animal farm owners/workers had better practice of antibiotic use. In this study, 46.2 percent of participants said they increased the antibiotic dose and frequency of administration as long as the animals showed no signs of recovery. This result indicated that the farmers took the initiative to change the antibiotic doses. Participants responded that they stopped giving antibiotics if animals appeared to be recovering at some point after using antibiotics, as previously reported by Adebowale, Adeyemo^38^, Gemeda, Amenu^30^ and Al-Mustapha, Adetunji^44^, which supports our findings.

Moreover 52.7% of the participants did not read the prospectus before using antibiotics and 57.1% did not go for laboratory test before choosing antimicrobial drugs for use of their animals. All of these abuses have been linked to an increase in antibiotic-resistant bacteria. More studies have found that non-compliance with antibiotic withdrawal periods is the leading cause of antibiotic drug residues in foods of animal origin^45–49^.

The majority of respondents believed that poor awareness on AMR, lack of rapid and effective diagnostic techniques, sub-standard quality of antibiotics and use of antimicrobials for animal growth promotion were the most important factors that contribute to increasing of antibiotic resistance. Although previous studies did not report results related to our study, some scholars reported on physicians', the general public's, and students' responses to factors that contribute to the rise of antibiotic resistance. According to previous reports, the most important factors for the spread and development of AMR were mis-/over-use of antimicrobials in animals^50^, lack of knowledge about prudent antibiotic use and antibiotic resistance^51^, irrational antibiotic use in humans^52^ and a lack of access to antibiotic susceptibility testing^53^.

According to the findings of this study, the majority of respondents believed that increasing the use of complementary treatments (herbs), developing rapid and effective diagnostic techniques, and educating prescribers and users on antimicrobial therapy were the most important measures that contribute to reducing antibiotic use and resistance. On the other hand, majority of the respondents perceived that reduction of antibiotic use for outpatient setting was not the main strategy that contribute to reduce of antibiotic resistance.

A study conducted in multi countries by^23^ reported that, most respondents agreed that Pharmaceutical companies should develop new antibiotics to address the problem of antibiotic resistance. Despite the development of a wide range of new potential antibiotic alternatives, including vaccines, bacteriophages, antimicrobial peptides, plant extracts, biofilm inhibitors, enzymes, and probiotics, it has been concluded that antibiotic resistance and tolerance in bacteria are natural evolutionary consequences and that prudent antibiotic use is the best option in the predictable future^54,55^.

## Conclusion

Our findings show that a significant number of animal farm owners/workers have inadequate knowledge and negative attitudes toward antibiotic use and resistance, as well as poor antibiotic use practices. Their KAP toward antibiotic use and resistance is linked to a number of socioeconomic factors, particularly their level of education. As a result, raising awareness about proper antibiotic use and antibiotic resistance is essential, and using alternative approaches should be encouraged. Furthermore, authorities should enforce restrictions on delivering, purchasing, and using antibiotics without a prescription in order to reduce antibiotic use and resistance. This study provides baseline data on KAP of animal farm owners/workers regarding antibiotic use and resistance, which may be useful to authorities in developing strategies to combat antibiotic resistance.

### Limitation of the study

The major limitation of this study was the relatively small number of participants selected from three cities of north western Ethiopia which might not reflect the real situation of KAP of animal farm owners/workers in Ethiopia as a whole. In addition, the cross-sectional study design can influence the cause and effect relationship of the predictor variables and the dependent variables (knowledge, attitude, and practice) of the animal farm owners/workers.

## Abbreviations

KAP: Knowledge, attitude, and practice
WHO: World Health Organization
FAO: Food and Agricultural Organization
AMU: Antimicrobial use
AMR: Antimicrobial resistance
CSA: Central Statistics Agency
ANOVAs: Analysis of variance
AMD: Antimicrobial drug
AM: Antimicrobial
SD: Standard deviation
MDR: Multidrug resistance
LMIC: Low- and middle-income countries
ABR: Antibiotic resistance
